# Isolation and Characterization of a Highly Mutated Chinese Isolate of Enterovirus B84 from a Patient with Acute Flaccid Paralysis

**DOI:** 10.1038/srep31059

**Published:** 2016-08-08

**Authors:** Huanying Zheng, Yong Zhang, Leng Liu, Jing Lu, Xue Guo, Hui Li, Hanri Zeng, Ling Fang, Wenbo Xu, Changwen Ke

**Affiliations:** 1 Guangdong Provincial Center for Disease Control and Prevention, No. 160, Qunxian Road, Panyu District, Guangzhou, China; 2 WHO WPRO Regional Polio Reference Laboratory, Key Laboratory of Medical Virology, National Health and Family Planning Commission of China, National Institute for Viral Disease Control and Prevention, Chinese Center for Disease Control and Prevention, Beijing, People’s Republic of China; 3 Guangdong Provincial Institution of Public Health, Guangzhou, China

## Abstract

Enterovirus B84 (EV-B84) is a newly identified serotype within the species Enterovirus B (*EV-B*). To date, only ten nucleotide sequences of EV-B84 are published and only one full-length genome sequence (the prototype strain) is available in the GenBank database. Here, a highly mutated EV-B84 (strain AFP452/GD/CHN/2004) was recovered from a patient with acute flaccid paralysis in the Guangdong province of China in 2004 making this the first report of EV-B84 in China. Sequence comparison and phylogenetic dendrogram analysis revealed high variation from the global EV-B84 strains (African and Indian strains) and frequent intertypic recombination in the non-structural protein region, suggesting high genetic diversity in EV-B84. The Chinese EV-B84 strain, apparently evolving independently of the other ten strains, strongly suggests that the EV-B84 strain has been circulating for many years. However, the extremely low isolation rate suggests that it is not a prevalent EV serotype in China or worldwide. This study provides valuable information about the molecular epidemiology of EV-B84 in China, and will be helpful in future studies to understand the association of EV-B84 with neurological disorders; it also helps expand the number of whole virus genome sequences of EV-B84 in the GenBank database.

Currently, human enteroviruses (EV) are classified into four species: *EV-A*, *EV-B*, *EV-C*, and *EV-D*^1^. With the wide use of molecular serotyping methods for EVs, the original serologically “untypable” strains have led to the discovery of a large number of new EV serotypes. Currently, the species *EV-B* constitutes 62 serotypes: coxsackievirus group B (CVB; serotypes 1–6), coxsackievirus group A (CVA; serotype 9), echovirus (serotypes 1–7, 9, 11–21, 24–27, 29–33), EV-B69, and the recently designated new EV serotypes EV-B73^2^, EV-B74–B75^3^, EV-B77–B78^4^,^5^,^6^, EV-B79–B88^7^,^8^,^9^,^10^, EV-B93^11^, EV-B97–B98^7^,^12^,^13^, EV-B100–B101^7^, EV-B106–B107^14^, and EV-B110–B113^15^. Enterovirus B84 (EV-B84) is a newly identified member of *EV-B*. To date, only one full-length genome sequence of EV-B84 (the prototype strain), from Cote d’Ivoire, is available in the GenBank database^7^.

The viruses of species *EV-B* are the most common viral cause of acute myocarditis, acute flaccid paralysis, and aseptic meningitis. They belong to the genus *Enteroviruses* in the family *Picornaviridae,* and order *Picornavirales*. Picornaviruses are small non-enveloped EVs comprising 60 copies of each of the capsid proteins VP4, VP2, VP3, and VP1, which enclose a positive-sense single-stranded RNA genome. The viral RNA (approximately 7450 nucleotides) contains a long open reading frame flanked by a 5′-untranslated region (UTR) and a 3′-UTR. The 5′-UTR is about 740 nucleotides in length and includes a secondary structure called the internal ribosome entry site^16^ which is involved in the replication and internal initiation of genomic RNA translation^17^. A single polyprotein translated from the RNA strand is first cleaved into three polyprotein precursors: P1, P2, and P3. P1 is processed to yield the four structural proteins, VP1–VP4, whereas P2 and P3 are precursors of the nonstructural proteins 2A–2C and 3A–3D, respectively.

Among the enterovirus serotypes, more than 60 are known human pathogens, even though most human enteroviral infections are asymptomatic or only result in mild disease, such as common cold. However, in some cases, enteroviruses are the most common viral cause of serious illnesses such as acute flaccid paralysis (AFP), acute myocarditis, aseptic meningitis, and neonatal sepsis-like disease^18^. In the present study, we report the complete genome sequence of an EV-B84 isolate that was recovered from a child aged 5 years with AFP in the Guangdong province of China in 2004. The virus was identified by comparison with enterovirus prototype strains and the recent enterovirus isolates that were partially characterized. The data presented here indicates that a highly mutated EV-B84 strain is circulating in China, making this the first report of EV-B84 and its probable association with acute flaccid paralysis.

## Results

### Molecular serotyping and primary characterization

Both RD (Human rhabdomyosarcoma) cells and HEp-2 (Human laryngeal carcinoma) cells support the growth of strain AFP452/GD/CHN/2004 (hereafter referred to as strain AFP452), and the appearance of cytopathic effects (CPEs) on RD cells is more rapid than on HEp-2 cells. This CPE positive isolate cannot be neutralized by a micro-neutralization test using a poliovirus-type specific rabbit polyclonal antiserum and a set of intersecting enterovirus antisera pools (Rijksinstituut Voor Volksgezondheid En Milieu; RIVM, The Netherlands)^19^. Therefore, it was considered as an “untypable” non-polio enterovirus (NPEV) and was further analysed by molecular methods.

**Figure 1:**
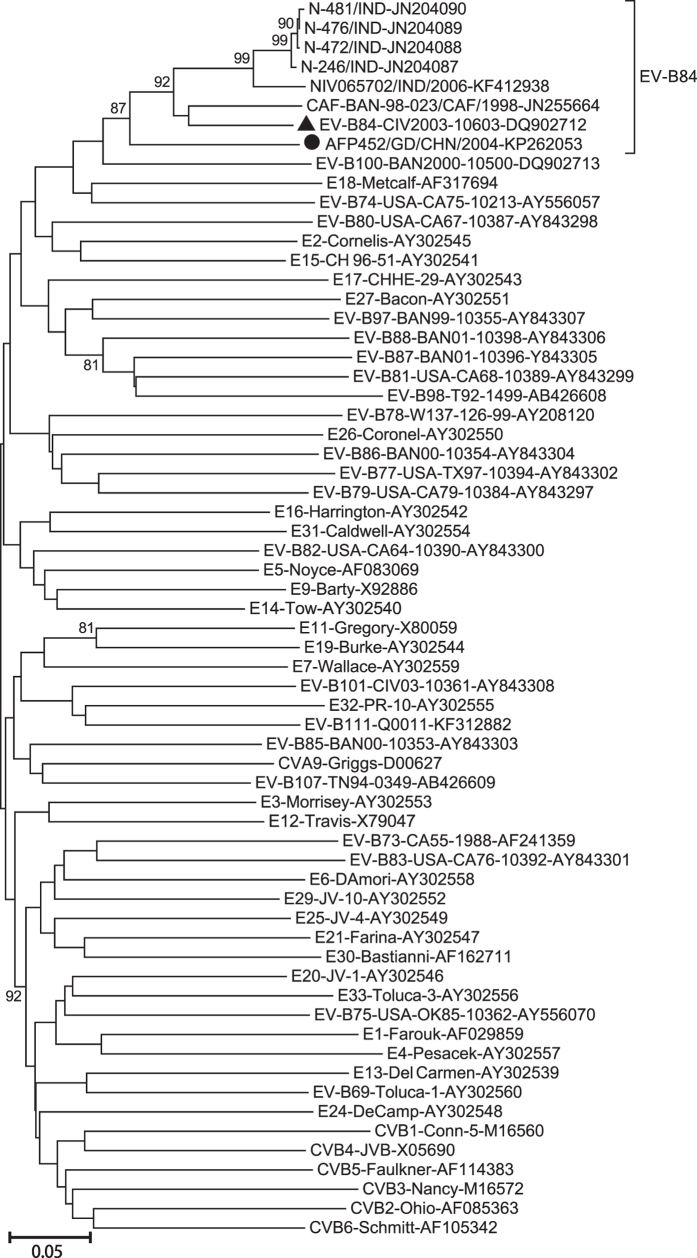
Phylogenetic analysis of the Guangdong strain collected in this study and all the EV-B prototype strains based on the entire *VP1* sequences. The Guangdong strain isolated in this study is indicated with a solid circle. The strain indicated by a triangle is the EV-B84 prototype strain. The tree was constructed using the Neighbour-Joining method and Kimura-two parameter model. The bootstrap support values were calculated for 1000 replicates and bootstrap support >80% are indicated for the main nodes.

RD cells were harvested after complete EV-like CPEs were observed, and the entire *VP1* region sequence was determined. Molecular serotyping of strain AFP452 based on the entire *VP1* sequence was performed using an online EV genotyping tool^20^, and the results indicated that strain AFP452 belongs to the EV-B84 serotype. Phylogenetic analysis based on the entire *VP1* coding regions of strain AFP542 and all the prototype *EV-B* strains available in the GenBank database was conducted (Fig. 1). As expected, all EV-B84 strains clustered together in the phylogenetic tree, with a bootstrap value of 87%, confirming the classification of these isolates as a single enterovirus serotype.

The sequence of the *VP1* coding region of strain AFP452 displayed 81.03% nucleotide and 94.48% amino acid identities with the EV-B84 prototype strain (strain CIV2003-10603/CIV/2003, GenBank number: DQ902712), confirming that these strains belong to the same serotype (the criteria are 75% VP1 nucleotide sequence and 88% amino acid identities within a serotype)^21^. In addition, it displayed 74.7–76.8% nucleotide and 88.3–89.7% amino acid identities with previously described EV-B84 strains. In contrast, it shared 67.70–77.24% nucleotide sequence identity in the *VP1* coding region with the other *EV-B* serotypes, and less than 52% identity with other Enterovirus species (*EV-A*, *EV-C*, *EV-D*).

### Full-length genome sequence of the Chinese EV-B84 strain

The full-length genome sequence of strain AFP452 was 7,421 nucleotides (nt) in length, encoding a polypeptide of 2,192 amino acids. The coding sequences were flanked by a non-coding 5′-UTR of 742 nt, a non-coding 3′-UTR of 103 nt, and a poly(A) tail composed of a long sequence of adenine nucleotides was added to the 3′ end of the genome. Analysis of the complete genome sequence of strain AFP452 showed that its genome was collinear with that of the prototype strain of EV-B84 (CIV2003-10603), except for two deletions at nt 120 and nt 121 in the 5′-UTR region and one deletion at nt 7325 and one insertion at nt 7343 in the 3′-UTR region. The overall base composition of the strain AFP452 genome was 28.07% A, 24.73% G, 23.39% C, and 23.81% U.

A comprehensive comparison of the nucleotide sequence and deduced amino acid sequence of the Chinese EV-B84 strain with that of the EV-B84 prototype strain (CIV2003-10603) and other prototype strains belonging to *EV-B* is shown in Table 1. The polypeptide cleavage sites were estimated using the complete genome sequence of the EV-B84 prototype strain, since the nucleotide sequences available for the distinct EV-B84 strains differ considerably. Overall, the complete genome sequence identity and the deduced amino acid sequence identity between the Chinese EV-B84 strain and the EV-B84 prototype strain were 79.97% and 95.35%, respectively.

### Phylogenetic and recombination analysis with EV-B prototype strains

**Figure 2:**
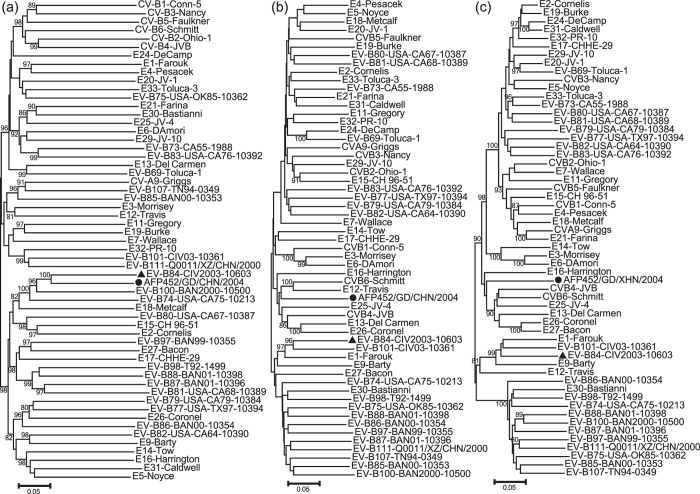
Phylogenetic relationships based on the *P1*, *P2*, and *P3* regions of enterovirus B (EV-B). Guangdong EV-B84 strain (indicated by a solid circle) and 55 other EV-B prototype strains were analysed by nucleotide sequence alignment using the Neighbour-Joining algorithms implemented in the MEGA 6.0 program. Numbers at the nodes indicate bootstrap support for that node (percentage of 1000 bootstrap replicates). The solid triangle indicates the EV-B84 prototype strain. Scale bars represent the genetic distance, and all panels have the same scale. (**A**) *P1* coding sequences; (**B**) *P2* coding sequences; and (**C**) *P3* coding sequences.

**Figure 3:**
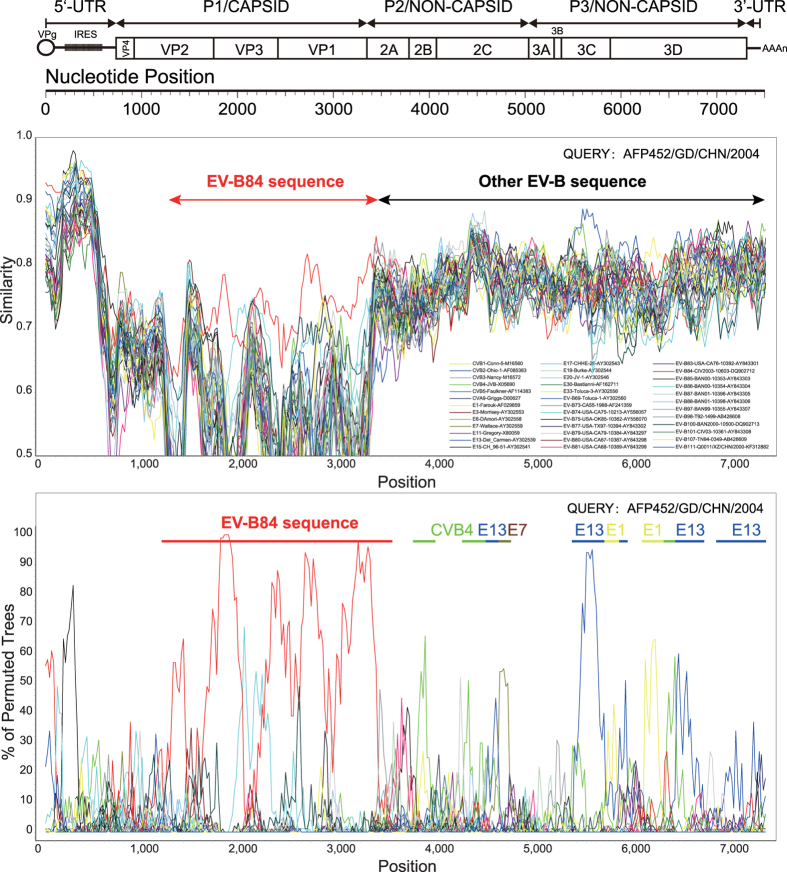
Recombination analyses of complete enterovirus A (*EV-A*) genomes. Similarity plot (**A**) and bootscanniSng analysis (**B**) of complete EV-B genomes using a sliding window of 200 nt moving in 20-nt steps. The Guangdong strain (AFP452) was used as a query sequence and is indicated in the lower right corner, and for each bootscan analysis, the names of the viruses in the query sequence are indicated in the upper right corner.

A phylogenetic tree based on the entire *P1*, *P2*, and *P3* coding regions of strain AFP452, and all the EV-B prototype strains (including EV-B84) available in the GenBank database was conducted (Fig. 2). Analysis of the phylogenetic tree suggested that the Chinese EV-B84 strain is monophyletic only in the capsid-coding (*P1*) region (Fig. 2A), whereas it grouped together with other enterovirus serotypes in EV-B such as Echovirus 13 in the nonstructural protein *P2* and *P3* coding regions (Fig. 2B,C). This suggests that recombination events occurred between Guangdong EV-B84 and other *EV-B* serotypes. To confirm the existence of recombination events in strain AFP542, similarity plot and bootscanning analyses were conducted with the other *EV-B* prototype strains (Fig. 3). The results revealed multiple recombination events between strain AFP542 and the EV-B strains at multiple sites in the *P2* and *P3* coding regions.

## Discussion

AFP case surveillance was established as part of the Global Polio Eradication Initiative in China since 1994^22^. As the earliest disease surveillance system in China, while monitoring the prevalence of polioviruses, NPEVs could also be detected simultaneously, and so the investigation of these viruses can provide valuable information on the molecular epidemiology of local EVs. Over the past 20 years or so, a large number of NPEVs were isolated and characterized in China, and the pathogen spectrum of EVs isolated from the AFP case surveillance were well studied in some provinces of China^23^,^24^,^25^.

Molecular serotyping methods based on sequence variation in the *VP1* region correlate with the classification based on antigenic properties^21^,^26^, and have recently replaced the neutralization test as the gold standard for enterovirus serotyping. Since the invention and application of molecular serotyping methods, a considerable number of new EV serotypes have been identified in many Chinese provinces, especially in Shandong^13^,^27^, Yunnan^14^, Xinjiang^28^,^29^, Tibet^3^,^15^,^30^, and Guangdong^31^. In this study, we report the full-length genome sequence of a recently identified novel serotype, EV-B84, which was also obtained from AFP case surveillance. To the best of our knowledge, this is the first report of EV-B84 in China.

The AFP patient was vaccinated with five doses of oral polio vaccine (OPV) after birth; and symptoms of acute flaccid paralysis appeared when he was five years old, residual paralysis still existed at 60 days after onset. Although only EV-B84, but no polioviruses or other viruses, were detected from the two stool samples collected from the patient, it was hard to conclude that EV-B84 was the causative agent in this AFP case under these circumstances, because no other epidemiological and laboratory evidence was obtained. Unfortunately, any other information cannot be achieved because this is a retrospective study. In addition, all ten EV-B84 strains available in the GenBank database (GenBank numbers: DQ902712, JX476198-JX476199, HQ662321, JN204087-JN204090, JN255664, and KF412938) were isolated from stool specimens of patients presenting with AFP, during the AFP patient surveillance activities in support of global polio eradication in different countries, including the prototype strain of EV-B84 (GenBank numbers: DQ902712). However, no confirmed correlation of EV-B84 with AFP has been described previously due to the limited number of viruses isolated. Further investigations including sero-epidemiology of this virus in populations might provide valuable information.

Sequence divergence is greatest in the *VP1* coding region due to evolution of a given enterovirus serotype. In contrast, the non-capsid regions do not correlate with enterovirus serotype due to frequent recombination within these regions, with recombination usually occurring among enterovirus serotypes within a species^32^,^33^. The Guangdong EV-B84 strain (AFP452) from the present study is no exception—it recombined with other *EV-B* viruses in the non-structural coding region, and as other numerous reports demonstrating *EV-B* recombination are available^30^,^34^,^35^, it is impossible to identify parental sequences for a studied genome. *EV-B* was found to be the predominant species in stool samples during AFP patient surveillance in China^23^,^24^. Hence, the possibility of co-infection with EV-B84 and other *EV-B* viruses is very large, and recombination events may have occurred subsequently.

There are currently ten EV-B84 sequences available in the GenBank database (at the end of 2015), and among them, only one (the prototype strain) is a full-length genome sequence. The ten EV-B84 strains available were isolated from two continents during 1998–2009, i.e., Africa (Cote d’Ivoire, and Central African Republic) and Asia (India), indicating that EV-B84 infection shows a global distribution. The substantial divergence between the *VP1* coding sequences and the existence of several recombination events between non-capsid sequences result in the formation of several clusters in phylogenetic analysis and suggest the circulation of distinct EV-B84 lineages in different countries. The existence of Chinese EV-B84 strain, apparently evolving along lineages independent of the other EV-B84 strains (African and Indian strains), strongly suggests that EV-B84 has been circulating for many years. However, the extremely low isolation rates suggest that it is not a prevalent EV serotype in China or even worldwide.

Strain AFP452 was isolated from a stool sample in 2004 during AFP patient surveillance activities in support of global polio eradication in the Guangdong province of China. The viral isolate was stored in a minor 40 °C freezer in Guangdong provincial polio laboratory for more than ten years. In order to study the antigenic properties of the virus, we inoculated the viral isolate on RD cell again to propagate the virus, but unfortunately the virus seemed to not survive, there may have been a problem with the storage conditions. In addition, an effective EV pathogen surveillance system is yet to be established in the mainland of China, and so any current information on EV-B84 or other new EV types in circulation is not available. Once this is in place, it is expected that monitoring trends in transmission of EV-B84 or other new EV types will be possible by molecular epidemiological studies over a wide area.

This study provides valuable information about the molecular epidemiology of EV-B84 in China, and will help future studies to understand the association of EV-B84 with neurological disorders; it also helps expand the number of virus genome sequences of EV-B84 in GenBank database.

## Material and Methods

### Sample collection and viral isolation

This study did not involve human participants or human experimentation; the only human materials used were stool samples collected from a child aged 5 years with AFP at the instigation of the Ministry of Health, P. R. of China, for public health purposes and written informed consent for the use of his stool samples was obtained from his parents. Samples were collected in accordance to guidelines of the Ministry of Health P. R. of China for public health purposes. This study was approved by the Ethics Review Committee of the Guangdong Center for Disease Control and Prevention.

The patient was a 5-year-old child with acute flaccid paralysis in Guangdong province in 2004. The flaccid paralysis symptoms appeared in September 24th, 2004, and without obvious fever. The paralysis mainly involves the double lower limbs of the patient, the leg muscle strength is zero and his two legs cannot move at all, and his double upper limb involvement is not obvious. The onset was not accompanied by diarrhea, dyspnea, neck rigidity, myodynia, and extremity sensory disturbance. Babinski’s sign is negative, deep tendinous reflect disappeared, and he was diagnosed as Guillain-Barre Syndrome in local hospital. Most unfortunately, paralysis residue was found during 60 days follow up.

Two stool samples, one collected on the day of illness onset and another on the subsequent day (with 24 h interval), were inoculated onto RD and HEp-2 cell lines. The virus isolate, strain AFP452/GD/CHN/2004, was not neutralized by intersecting enterovirus antisera pools (RIVM), performed according to standard procedures^19^.

### Molecular serotyping

For molecular serotyping, viral RNA was extracted from the viral isolate by using a QIAamp Viral RNA Mini Kit (Qiagen. Germany) and was stored at −80 °C until use. Reverse transcription polymerase chain reaction (RT-PCR) was performed to amplify the *VP1* coding region using PrimeScript One Step RT-PCR Kit Ver.2 (TaKaRa, Dalian, China) with primer pairs E012 as forward and E011 as reverse^36^. The PCR products were purified using the QIAquick PCR purification kit (Qiagen, Germany) and subjected to nucleotide sequencing. Sequencing was performed in both directions using an ABI 3130 Genetic Analyzer (Applied Biosystems, USA), and every nucleotide position was sequenced at least once from each strand. The sequences were analysed with the Basic Local Alignment Search Tool server at the National Center for Biotechnology Information and EV serotype was determined according to a previously described molecular serotyping method^20^.

### Full-length genome amplification

One microliter (200U) of SuperScript II ribonuclease H- reverse transcriptase (invitrogen, USA) was used to produce single stranded cDNA from 5 μL of purified viral RNA. The cDNA syntheses were primed with 7500A and E011 (Table 2), respectively, and performed at 42 °C for 2 h, followed by 60 °C for 15 min to inactivate the enzyme. Finally, RNA in an RNA:DNA hybrid was specifically degraded with 1 μL ribonuclease H (Promega, USA) at 37 °C for 30 min. Two long-distance PCR amplifications were performed by using the TaqPlus Precision PCR system (Stratagene, USA), which consists of a blend of Stratagene cloned Pfu DNA polymerase (proof reading) and Taq2000 DNA polymerase (non-proof reading). Reactions contained 5 μL of cDNA (see above), 0.1 mM of each dNTP, 10 μL of TaqPlus buffer, 1.0 ng/μL of the forward (0001S48 or 008) and reverse (011 or 7500A) primers (Table 2), and 5 U of TaqPlus enzyme in a 100 μL reaction. Amplification was performed by 35 cycles through temperature levels of 94 °C (30 s), 60 °C (30 s), and 72 °C (6 min), followed by another two temperature levels of 94 °C (1 min) and 72 °C (20 min). The primers used for sequencing of the full-length genome were designed by a “primer-walking” strategy and are listed in Table 2. 5′ segment sequences were determined by using the 5′ rapid amplification of cDNA ends (RACE) core set (Takara Biomedicals) according to the manufacturer’s instructions.

### Bioinformatics analysis

Raw sequence data were assembled using the Sequencher software (version 4.0.5, Gene Codes Corporation). Multiple sequence alignments and distance matrices were generated using the MEGA program (version 6.0, Sudhir Kumar, Arizona State University, Tempe, Arizona, USA)^36^. The phylogenetic dendrograms were generated by the Neighbour-Joining method using the Kimura-2 parameter model for aligned nucleotide sequences in the MEGA program. The tree topology was obtained by majority rule consensus among 1000 bootstrap replicates shown as percentages, and bootstrap values greater than 80% were considered to be statistically significant for grouping. Similarity plot and bootscanning analysis for potential recombination were performed using the Simplot program (version 3.5.1)^37^.

### Nucleotide sequence accession number

The nucleotide sequence of the complete genome of the Chinese EV-B84 strain AFP452/GD/CHN/2004 has been deposited in the GenBank database (Accession No. KP262053).

## Additional Information

**How to cite this article**: Zheng, H. *et al.* Isolation and Characterization of a Highly Mutated Chinese Isolate of Enterovirus B84 from a Patient with Acute Flaccid Paralysis. *Sci. Rep.* **6**, 31059; doi: 10.1038/srep31059 (2016).

## Figures and Tables

**Table 1:** Nucleotide sequence and deduced amino acid sequence identities between the Guangdong EV-B84 strain (AFP452), the EV-B84 prototype strain, and other prototype strains belonging to EV-B.

Region	% nucleotide identity (% amino acid identity)	
	EV-B84 strain AFP452	
	Prototype of EV-B84	Prototypes of other EV-B
5′ UTR	90.42	82.15–90.16
VP4	79.71 (88.41)	68.12–82.61 (76.81–95.65)
VP2	78.59 (95.83)	71.16–78.21 (78.03–89.77)
VP3	78.25 (95.42)	65.51–74.38 (68.33–85.00)
VP1	81.03 (94.48)	67.70–77.24 (72.76–90.34)
2A	81.11 (93.33)	73.78–82.00 (84.67–95.33)
2B	78.11 (94.95)	75.76–81.82 (92.93–97.98)
2C	80.45 (98.48)	79.53–84.19 (96.35–98.78)
3A	76.78 (96.63)	75.66–83.90 (91.01–98.88)
3B	84.85 (100.0)	74.24–87.88 (90.91–100.0)
3C	79.78 (97.27)	77.05–86.16 (95.08–99.45)
3D	80.66 (97.40)	77.63–83.62 (95.24–98.27)
3′ UTR	86.41	84.47–94.17

**Table 2:** PCR and sequencing primers.

Primer	Nucleotide position (nt)	Primer sequence	Orientation	Reference
0001S48^a^		GGGGACAAGTTTGTACAAAAAAGCAGGCTTTAAAACAGCTCTGGGGTT	Forward	38
AFP452-1446A	1427–1446	TGAACCACCTTGCCTGTAAC	Reverse	This study
AFP452-1888A	1869–1888	CACAACGGAATCAACCTCAG	Reverse	This study
AFP452-2522A	2503–2522	CTGGGATATGGGTGGAGTTG	Reverse	This study
AFP452-2584A	2565–2584	TGTTTGCAATGTGTCACCAG	Reverse	This study
008	2411–2430	GCRTGCAATGAYTTCTCWGT	Forward	21
011	3389–3408	GCICCIGAYTGITGICCRAA	Reverse	21
AFP452-3247S	3247–3267	CATCACTGACGAACGAACTGA	Forward	This study
AFP452-3959S	3959–3978	ACAGTGACTGCCACTCTAGC	Forward	This study
AFP452-4669S	4669–4688	AATGGCCGCATTGGAGGAGA	Forward	This study
AFP452-5298S	5298–5317	AGTTGTTCGCAGGATTCCAG	Forward	This study
AFP452-5919S	5919–5938	TTAATGATGAGCAAGGAGAG	Forward	This study
AFP452-6638S	6638–6657	GGATATGATGCCAGCTTGAG	Forward	This study
7500A^a^		GGGGACCACTTTGTACAAGAAAGCTGGG(T)_24_	Reverse	38

^a^The primer pairs 0001S48/011 and 008/7500A are suggested for long distant PCR. The expected amplicons from these are 3.40 kb and 4.99 kb, respectively.
